# Preliminary exploration of prediction model and nomograph drawing of Doppler peak systolic velocity ratio, peak systolic velocity 1 and peak systolic velocity 2 of ophthalmic artery in pregnant women for small for gestational age infants

**DOI:** 10.3389/fmed.2025.1598587

**Published:** 2025-08-20

**Authors:** Yanxia Yang, Huifang Liu, Siqi Yang, Qianyi Huang, Shaohui Chen

**Affiliations:** ^1^Department of Ultrasound, The Sixth Affiliated Hospital of Jinan University, Dongguan, China; ^2^Department of Medical Laboratory, The Sixth Affiliated Hospital of Jinan University, Dongguan, China; ^3^Department of Obstetrics, The Sixth Affiliated Hospital of Jinan University, Dongguan, China

**Keywords:** ophthalmic artery Doppler, PSV ratio, PSV1, PSV2, small for gestational age infants, prediction models

## Abstract

**Objective:**

To explore the predictive value of peak systolic velocity (PSV) ratio, PSV1 and PSV2 of ophthalmic artery Doppler in pregnant women for small for gestational age (SGA) infants and to construct a nomogram prediction model.

**Methods:**

A total of 201 pregnant women who visited our hospital from March 2022 to June 2024 were selected as the research subjects, and their clinical data and ophthalmic artery Doppler parameters were collected. The data were randomly divided into a training set (*n* = 295) and a verification set (*n* = 126) in a 7:3 ratio. The independent risk factors for SGA were screened by univariate and multivariate Logistic regression analysis, and the nomogram model was constructed. The model calibration degree, prediction efficiency and clinical value were evaluated.

**Results:**

The incidence of SGA in the training and validation sets was 25.42 and 25.31%, respectively. Multivariate Logistic regression analysis showed that PSV ratio of ophthalmic artery, PSV1, PSV2, Pulse index 1 (PI1), PI2, and gestational week were the independent risk factors for SGA (all *p* < 0.05). The C-index of the nomogram model in the training set and the verification set was 0.858 and 0.835, respectively, the area under the ROC curve (AUC) was 0.858 (95% *CI*: 0.804–0.912) and 0.835 (95% *CI*: 0.734–0.936), and the sensitivity and specificity were 0.887, 0.747 and 0.636 and 0.833, respectively. The calibration curve showed good agreement between the predicted and actual values, respectively, which was good by the Hosmer-Lemeshow test. The analysis of decision curve showed that the model had high clinical application value in the range of threshold probability 0.10–0.80.

**Conclusion:**

The nomogram model based on PSV ratio of ophthalmic artery Doppler, PSV1, and PSV2 has good prediction performance for SGA, and provides a new tool for early clinical recognition of high-risk pregnant women with SGA. However, the accuracy and applicability of the model still need to be further verified in the multi-center large sample study.

## 1 Introduction

Small for Gestational Age (SGA) is defined as the newborn whose birth weight is lower than the 10th centile of the mean weight for gestational age. Its perinatal morbidity and mortality are high, and it may face the risks of growth retardation and metabolic diseases in the long term (1). Accurately predicting the occurrence of SGA is of great significance for early intervention and improving the prognosis of mother and infant. Ophthalmic artery, a maternal vessel branching from the internal carotid artery, Doppler ultrasound as a non-invasive and repeatable imaging method, its hemodynamic parameters may be associated with fetal growth and development through maternal-placental circulation interactions. Studies have shown that its parameters are closely related to fetal growth restriction. However, there are few studies on the SGA prediction model based on multi-parameters of ophthalmic artery Doppler (2). In addition, the clinical significance of novel parameters such as first peak systolic velocity (PSV1) and second peak systolic velocity (PSV2) in the assessment of maternal ophthalmic artery hemodynamics and their association with SGA has not been completely clarified (3). At present, there are few studies on joint multi-parameter prediction model in China and abroad, and it is lack of verification based on large sample queue. This study was intended to include single-fetus pregnant women who were archived in the obstetrics department of our hospital from March 2022 to June 2024 through a retrospective cohort study, and to collect the ophthalmic artery Doppler ultrasound data at ≥28 weeks of gestation. The study will focus on analyzing the correlation between PSV ratio, PSV1, PSV2 and the centile of neonatal birth weight, screening the independent risk factors using Logistic regression model, and verifying the stability of the model by Bootstrap method. The finally constructed nomogram prediction model will integrate clinical indicators with ultrasound parameters, aiming to achieve layered early warning for SGA. The innovation of this study lies in the combination of ophthalmic artery blood flow parameters and placental function indicators for the first time, which provides a quantifiable prediction tool for clinical practice and is expected to promote the change of SGA prevention and control strategy from empirical intervention to precise management (4).

## 2 Materials and methods

### 2.1 Subjects

A total of 421 pregnant women who visited the obstetrics department of our hospital from March 2022 to June 2024 were selected as the research subjects. Inclusion criteria: ① single pregnancy; ② Gestation week ≥28 weeks (determined by last menstrual period and confirmed by first-trimester ultrasound biometry); ③ Doppler examination of ophthalmic artery was performed before delivery; ④ Sign informed consent form. Exclusion criteria: ① Patients with severe pregnancy complications (such as preeclampsia and gestational diabetes mellitus); ② The fetus has structural deformity or chromosome abnormality; ③ Smoking and alcohol abuse during pregnancy; ④ those with incomplete data. The patients were divided into a training set (*n* = 295) and a verification set (*n* = 126) using random number table. This study was approved by the Hospital Ethics Committee.

### 2.2 Data collection

Demographic data including maternal age, pre-pregnancy BMI (calculated as kg/m^2^), and ethnicity (all participants in this study were Han Chinese) were collected. The ophthalmic artery was examined using a Philips EPIQ 5 color Doppler ultrasound system (Philips Healthcare, Amsterdam, the Netherlands) equipped with a 7.5 MHz linear transducer. The patient was in a supine position after a 5-min rest period with eyes closed (5). The artery was identified medial and superior to the optic nerve via color flow imaging, and pulsed-wave Doppler was applied with a 2 mm sample gate, 125 kHz pulse repetition frequency, 50 Hz high-pass filter, and insonation angle <20 degrees. Three to four consecutive waveforms were recorded for each eye, and parameters were averaged from two measurements per eye to minimize variability (6). Specifically, the following parameters were measured: ① PSV1: The highest velocity of blood flow during systole in the ipsilateral ophthalmic artery, reflecting the arterial blood flow velocity during ventricular contraction.; ② End diastolic flow velocity (EDV1): The lowest velocity of blood flow during diastole in the ipsilateral ophthalmic artery, reflecting the residual blood flow during ventricular relaxation. ③ Pulse index 1 (PI1): Calculated as (PSV1 − EDV1)/mean flow velocity, which reflects the resistance to blood flow and is influenced by vascular compliance and peripheral resistance ④ Resistance index 1(RI1): Calculated as (PSV1 − EDV1)/PSV1, a marker of vascular resistance; higher values indicate increased resistance. For each ophthalmic artery, three to four consecutive waveforms were recorded, and parameters were derived from the same artery: PSV1 (first peak systolic velocity), PSV2 (second peak systolic velocity), EDV1/EDV2 (corresponding end diastolic velocities), PI1/PI2 (pulsatility indices), and RI1/RI2 (resistance indices), and the PSV ratio (PSV1/PSV2) was calculated. Neonatal birth weight, length, head circumference and other indicators were recorded, and whether SGA was determined according to the gestational age and gender reference standard growth curve.

### 2.3 Diagnostic criteria

The SGA was diagnosed as having a birth weight less than the 10th percentile of the weight of a gestational age, sex-matched newborn (using a Fenton growth curve).

### 2.4 Estimation of sample size

Sample size calculation was performed using PASS 15.0 software based on previous literature and preliminary data. Considering the incidence of SGA as approximately 25% in the target population, with a significance level (*α*) set at 0.05 (two-tailed) and statistical power (1-*β*) at 80%, the minimum required sample size was estimated to be 385 cases. Accounting for potential loss to follow-up (10%), a total of 421 pregnant women were enrolled in this study, which met the statistical requirements.

### 2.5 Statistical analysis

SPSS25.0 and R4.0.3 software were used for data analysis. When the measurement data conforms to the normal distribution, the measurement data were expressed as mean ± standard deviation (SD), and the comparison between the two groups is made by *t*-test. M (Q1, Q3) is used when it does not conform to the normal distribution, and Mann–Whitney *U* test is used for comparison between groups. The count data were expressed as number of cases and percentage (*n*, %), and the comparison between groups was performed by *χ* test. Logistic regression analysis was used to screen the independent risk factors affecting the curative effect of treatment. Application of r software to construct nomograph prediction model based on independent risk factors. The receiver operating characteristic (ROC) curve was used to evaluate the prediction performance of the model, and the Area Under Curve (AUC) and 95% Confidence Interval (CI) were calculated. A calibration curve was used to evaluate the consistency of the model predictions with the actual observations. *p* < 0.05 was considered as the difference with statistical significance.

## 3 Results

### 3.1 Comparison of general clinical characteristics between training set and verification set

There was no significant difference in general clinical characteristics such as age, gestational week, pre-pregnancy BMI, history of hypertension and diabetes between the two groups (all *p* > 0.05) (Table 1).

**Table 1:** Comparison of general clinical features between training set and validation set.

Index		Training set (*n* = 295)	Validation set (*n* = 126)	Statistical values	*P*
Age (years)		28.89 ± 3.25	28.49 ± 3.35	1.146	0.253
Gestational weeks		37.88 ± 1.44	38.01 ± 1.23	0.885	0.377
Pre-pregnancy BMI (kg/m^2^)		23.82 ± 2.63	24.16 ± 2.56	1.224	0.222
Uterine artery PI		1.75 ± 0.25	1.71 ± 0.26	0.371	0.711
Body weight gain during pregnancy (kg)		11.61 ± 2.82	11.73 ± 3.05	0.391	0.697
History of hypertension	Have	32(10.85)	16(12.69)	0.299	0.584
	Without	263(89.15)	110(87.31)		
History of diabetes	Have	17(5.76)	13(10.32)	2.768	0.096
	Without	278(94.24)	113(89.68)		
Family history of disease	Have	40(13.56)	26(20.64)	3.344	0.068
	Without	255(86.44)	100(79.36)		
PSV ratio		1.15 ± 0.24	1.14 ± 0.25	0.387	0.699
PSV1 (cm/s)		33.82 ± 4.96	32.93 ± 4.72	1.711	0.088
PSV2 (cm/s)		29.54 ± 4.55	30.21 ± 5.01	1.342	0.181
PI1		1.82 ± 0.31	1.79 ± 0.30	0.918	0.359
RI1		0.71 ± 0.09	0.70 ± 0.08	1.078	0.282
PI2		1.72 ± 0.28	1.73 ± 0.35	0.311	0.756
RI2		0.72 ± 0.09	0.71 ± 0.08	1.078	0.282

### 3.2 Training set SGA single factor analysis of risk factors

In the training set, 75 cases (25.42%) of SGA31 were diagnosed. Univariate analysis showed that there were significant differences in Gestational weeks, PSV ratio, PSV1, PSV2, PI1 and PI2of ophthalmic artery between the SGA group and the non-SGA group (all *p* < 0.05) (Table 2).

**Table 2:** Univariate analysis of risk factors for SGA in training set.

Index		SGA group (*n* = 75)	Non-SGA group (*n* = 220)	Statistical values	*P*
Age (years)		29.21 ± 3.55	28.49 ± 3.15	1.654	0.099
Gestational weeks		37.48 ± 1.62	38.11 ± 1.43	3.183	0.002
Pre-pregnancy BMI (kg/m^2^)		23.52 ± 3.03	24.06 ± 2.71	1.445	0.149
Uterine artery PI		1.79 ± 0.35	1.72 ± 0.28	1.749	0.081
Body weight gain during pregnancy (kg)		11.21 ± 2.52	11.83 ± 3.15	1.544	0.124
History of hypertension	Have	12(16.00)	20(9.09)	2.761	0.096
	Without	63(84.00)	200(90.91)		
History of diabetes	Have	7(9.33)	10(4.54)	1.562	0.211
	Without	68(90.67)	210(95.46)		
Family history of disease	Have	10(13.33)	30(13.64)	0.004	0.947
	Without	65(86.67)	190(86.36)		
PSV ratio		1.25 ± 0.24	1.04 ± 0.23	6.625	0.001
PSV1(cm/s)		35.62 ± 5.26	31.93 ± 4.82	5.592	0.001
PSV2(cm/s)		28.44 ± 4.15	30.51 ± 5.03	3.211	0.002
PI1		1.89 ± 0.32	1.76 ± 0.28	3.345	0.001
RI1		0.72 ± 0.10	0.69 ± 0.12	1.946	0.053
PI2		1.76 ± 0.29	1.61 ± 0.25	4.303	0.001
RI2		0.72 ± 0.11	0.69 ± 0.12	1.909	0.057

### 3.3 Multivariate logistic regression analysis

SGA was used as the dependent variable (1 = Yes, 0 = No), and the variable *p* < 0.05 in the single factor analysis was used as the independent variable for Multivariate Logistic regression analysis. The results showed that PSV ratio of ophthalmic artery, PSV1, PSV2, PI1, PI2 and gestational week were the independent risk factors for SGA (all *p* < 0.05) (Table 3).

**Table 3:** Results of multivariate logistic regression analysis.

Project	*B*	Standard error	Wald	*P*	OR	95% confidence interval
PSV ratio	3.430	0.747	21.080	0.001	30.867	7.139–133.460
PSV1	0.138	0.034	16.368	0.001	1.148	1.074–1.227
PSV2	−0.072	0.035	4.236	0.040	0.931	0.869–0.997
PI1	1.706	0.565	9.110	0.003	5.506	1.819–16.669
PI2	2.019	0.616	10.739	0.001	7.533	2.251–25.204
gestational week	−0.318	0.108	8.668	0.003	0.728	0.589–0.899

### 3.4 Construction of nomogram prediction model

**Figure 1:**
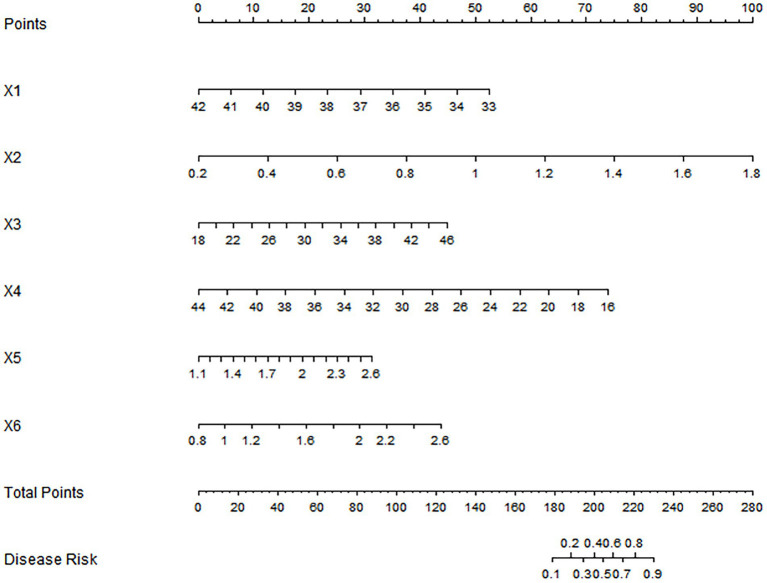
Nomogram model for predicting SGA. x1: PSV ratio; x2: PSV1, x3: PSV2, x4: PI1, x5: PI2, x6: gestational week.

Based on the independent risk factors determined by multivariate Logistic regression analysis, a nomogram model for predicting SGA was constructed. Each risk factor was given corresponding score according to its regression coefficient, and the total score corresponding to the prediction probability (Figure 1).

### 3.5 Evaluation and validation of nomogram model

**Figure 2:**
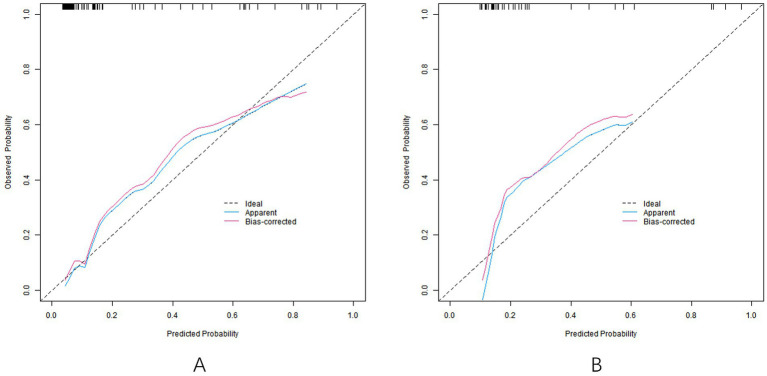
Calibration curve [calibration curves of models in training set **(A)** and validation set **(B)**].

**Figure 3:**
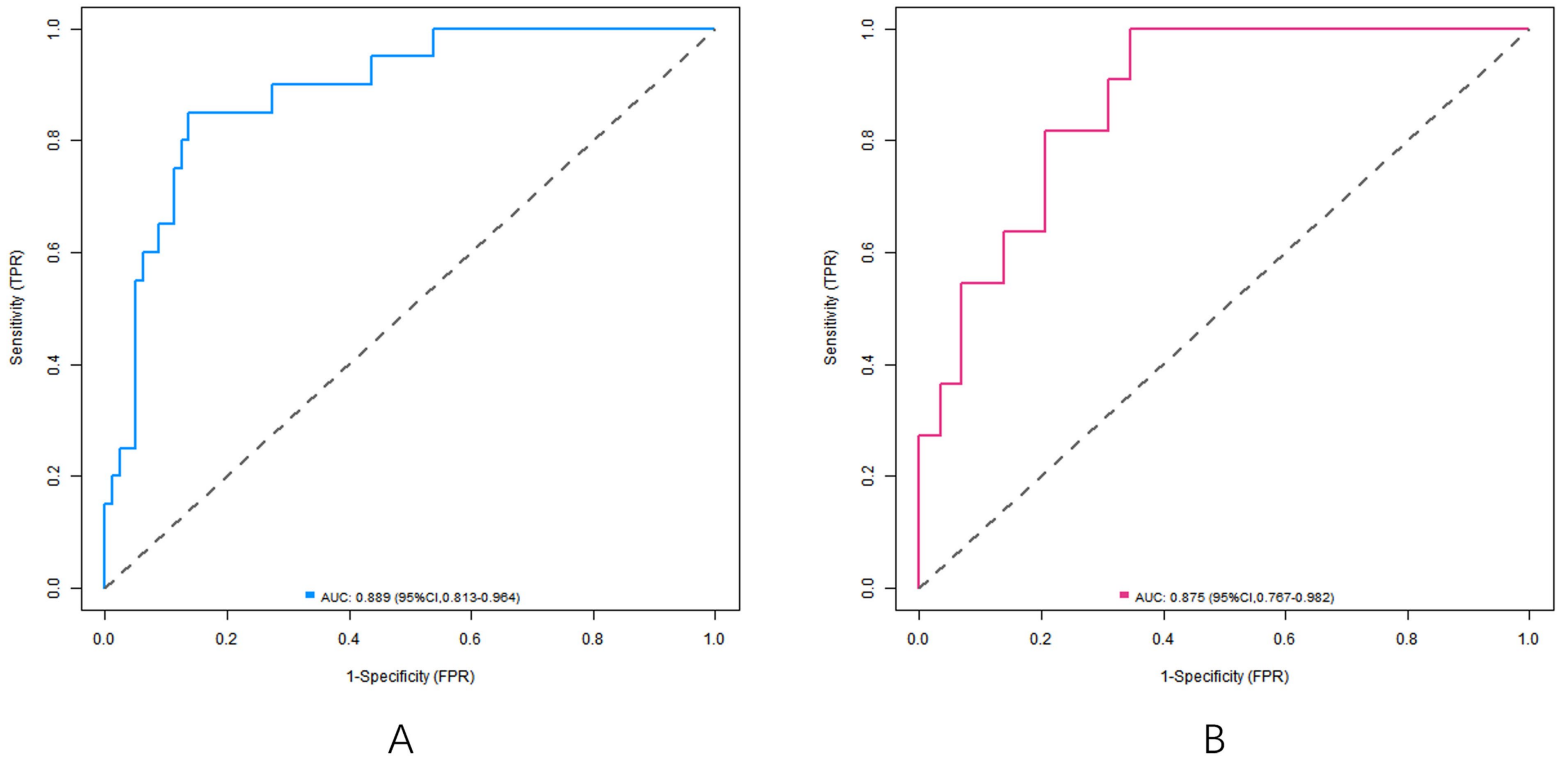
ROC curve [the ROC curve of the model in the training set **(A)** and the ROC curve of the model in the verification set **(B)**].

In the training set, the C-index of the nomogram model was 0.858, the Hosmer-Lemeshow test *p* = 0.692, indicating that the model fitted well. The ROC curve showed an AUC of 0.858 (95% *CI*: 0.804–0.912), a sensitivity of 0.887, and a specificity of 0.747. In the validation set, C-index was 0.835, Hosmer-Lemeshow test *p* = 0.804, AUC was 0.835 (95% *CI*: 0.734–0.936), sensitivity was 0.636, and specificity was 0.833. The calibration curve and ROC curve are shown in Figures 2, 3, respectively.

### 3.6 Decision curve analysis

**Figure 4:**
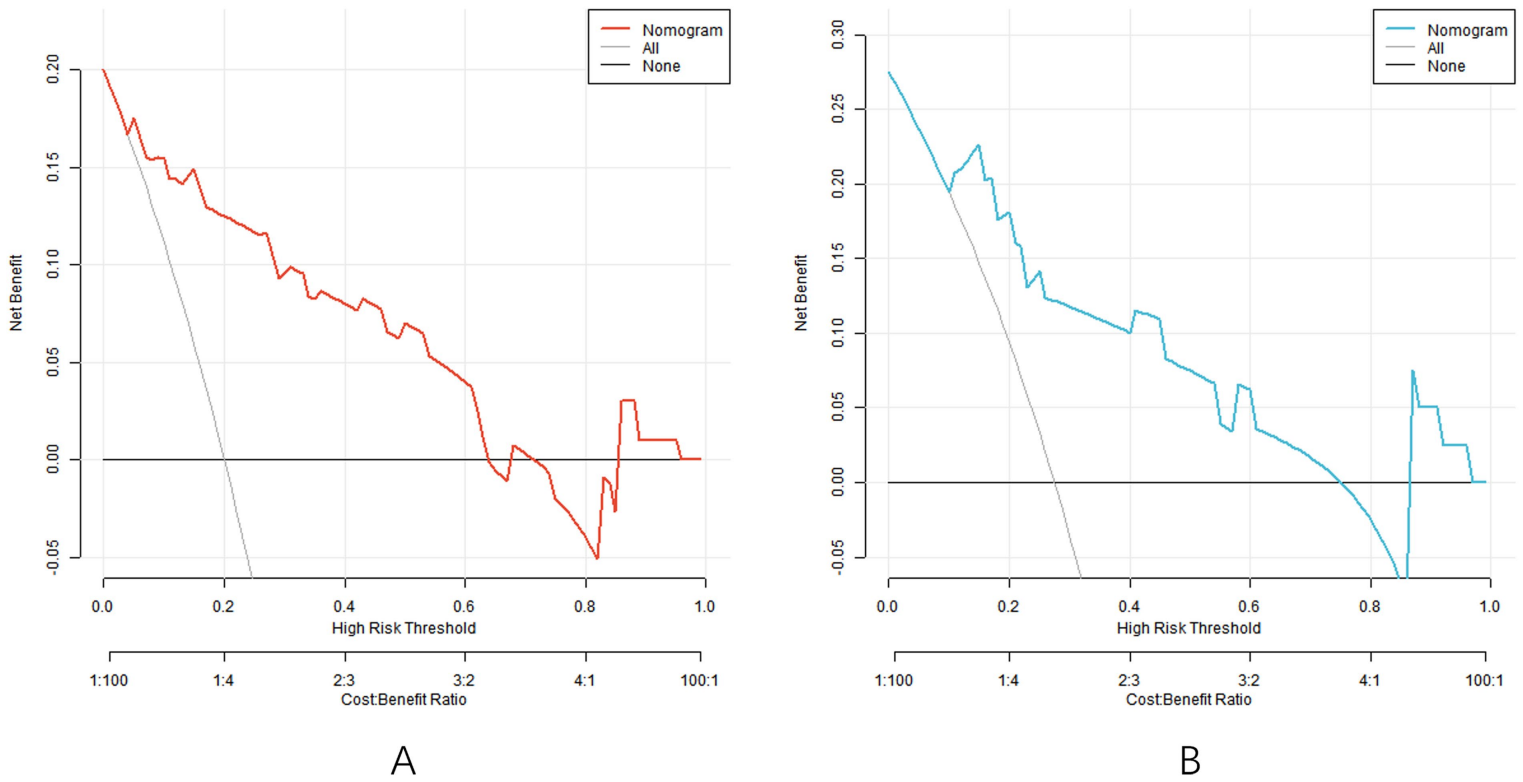
Decision curve [the decision curve of the model in the training set **(A)** and the decision curve of the model in the validation set **(B)**].

Decision curve analysis showed that when the threshold probability was within the range of 0.10–0.80, the nomogram model had high clinical application value for predicting SGA (Figure 4).

## 4 Discussion

The SGA is an important perinatal complication, and its incidence accounts for about 10% of the total number of neonates. It is not only associated with short-term risks such as fetal distress, hypothermia and hypoglycemia, but also may increase the risk of long-term health problems such as metabolic syndrome and cardiovascular disease in adulthood (7). Accurate prediction of SGA is of great significance for optimizing clinical intervention strategies and improving maternal and infant outcomes. At present, clinically commonly used prediction methods of SGA include fetal biometrics (such as biparietal diameter, abdominal circumference, and femoral length), maternal serological indicators (such as human chorionic gonadotropin and placental growth factor) and Doppler ultrasound to assess fetal hemodynamics (such as umbilical artery and uterine artery resistance index). However, the prediction efficiency of a single indicator is limited, and the joint application between different indicators has not yet formed a unified standard (8).

Ophthalmic artery is a branch of internal carotid artery, and its hemodynamic parameters can reflect fetal cerebrovascular resistance and blood flow redistribution (9). Previous studies have shown that in the case of fetal growth restriction (FGR), the flow resistance of the ophthalmic artery is decreased, manifested as decreased PI and RI, and increased PSV, which may be related to the “brain protection effect” initiated by the fetus to ensure blood supply to the brain (10). However, existing studies mostly focus on the association between single eye artery parameters and FGR, and there are few reports on the establishment of SGA prediction model using multiple eye artery parameters in combination. The purpose of this study was to explore the predictive value of PSV ratio, PSV1, PSV2, PI1, PI2 and gestational week in ophthalmic artery for SGA, and to construct a nomogram model, so as to provide a new tool for early clinical identification of high-risk pregnant women.

In this study, multivariate Logistic regression analysis showed that PSV ratio of ophthalmic artery (PSV1/PSV2) was the strongest independent risk factor for SGA (*OR* = 30.867), and its prediction efficiency was significantly higher than that of other parameters (11). This suggests that abnormal systolic flow dynamics in maternal ophthalmic artery may be linked to SGA.

From the perspective of mechanism, blood flow abnormalities in unilateral ophthalmic artery may reflect local vascular development or dysfunction, thus affecting the overall fetal blood flow distribution. Ophthalmic artery, a branch of the maternal internal carotid artery, primarily supplies the maternal eye and orbital structures. Its hemodynamic changes during pregnancy may reflect maternal cerebrovascular adaptation, which indirectly affects fetal growth by influencing uterine and placental perfusion. When the PSV of a unilateral ophthalmic artery increases, it may mean decreased vascular resistance or increased blood flow on that side, and this local abnormality may interfere with blood flow balance in the fetus, resulting in uneven delivery of nutrients and oxygen, thereby affecting fetal growth (12). Blood flow asymmetry may also indicate occult vasculopathy or placental insufficiency in the fetus (13). Placental insufficiency is one of the important causes of SGA and affects the material exchange between mother and fetus. The asymmetry of blood flow in the ophthalmic artery may be an early signal of placental insufficiency, indicating that the fetus redistributes blood flow to maintain blood supply to vital organs. The long-term existence of this compensatory mechanism may lead to fetal growth restriction (14). This study results are complementary to previous studies’ conclusion that unilateral ophthalmic artery parameters can predict FGR. Previous studies have mostly focused on single parameter of unilateral ophthalmic artery, such as PSV1 or PI1. However, in this study, the introduction of PSV ratio further revealed the importance of the difference in blood flow between the two ophthalmic arteries, thus providing a more comprehensive perspective for clinical evaluation. Univariate analysis showed that PSV1 in the SGA group was significantly higher than that in the non-SGA group (35.62 ± 5.26 vs. 31.93 ± 4.82 cm/s cm/s), while PSV2 was significantly reduced (28.44 ± 4.15 vs. 30.51 ± 5.03 cm/s cm/s). Multivariate regression further confirmed that both increased PSV1 (*OR* = 1.148) and decreased PSV2 (*OR* = 0.931) were independent risk factors. This paradox may be related to the compensatory mechanism of fetal hemodynamics. When placental function decreases, the fetus maintains blood supply to the brain by increasing cardiac output and reducing peripheral resistance (such as cerebrovascular dilatation), which leads to an increase in PSV1 in the ophthalmic artery. Because after cerebrovascular expansion, blood flow resistance is reduced, blood flow velocity is accelerated, so that the PSV1 increased. However, a decrease in PSV2 may reflect insufficient compensatory regulation of the contralateral ophthalmic artery, or suggest a broader vascular resistance abnormality. Specifically, when one ophthalmic artery increases blood flow by dilating blood vessels, the opposite ophthalmic artery may not compensate equally effectively, resulting in a relative decrease in blood flow velocity (15). In addition, this widespread vascular resistance abnormality may indicate that there is a problem with the circulatory system of the fetus as a whole that cannot efficiently distribute blood flow, thereby further affecting the growth and development of the fetus (16). PI is an important indicator reflecting vascular resistance. In this study, PI1 and PI2 of SGA group were significantly higher than those of non-SGA group, indicating that fetal ophthalmic artery resistance was increased. This result seems to contradict the theory of “brain protection effect.” According to the theory of “brain protection effect,” in the case of hypoxia or malnutrition of the fetus, the cerebral vessels will expand to increase cerebral blood flow and thus reduce the PI value. However, the increased PI value in this study may be due to the increased systemic vascular resistance of SGA fetus in the late stage of disease progression, which covered up the manifestations of local cerebrovascular dilatation. With the persistent existence of hypoxia and malnutrition in the fetus, the systemic blood vessels of the fetus will undergo adaptive changes, leading to a general increase in vascular resistance. This increase in systemic vascular resistance may outweigh the effects of local cerebrovascular dilatation, resulting in an increase in the PI value of the ophthalmic artery. In addition, the increased PI value may be related to vascular remodeling caused by chronic hypoxia in the fetus. The long-term hypoxia environment will promote changes in the structure of the vascular wall, increase vascular resistance, and further aggravate the fetal growth restriction (17). The study found a 30% reduction in SGA risk (*OR* = 0.728) for each additional week of gestation. This result is consistent with clinical common sense, as insufficient gestational weeks are one of the direct causes of SGA (18). The growth of the fetus in the uterus needs enough time, and as the gestational weeks increase, the fetus has more time to obtain nutrition and oxygen from the mother, thus promoting growth and development. Notably, pregnant women ≥28 weeks were included in this study, and gestational week still showed independent predictive value in the multivariate model, suggesting that accurate assessment of gestational week was important for risk stratification of SGA even in late pregnancy. In clinical practice, accurate assessment of gestational weeks can help doctors to detect abnormal fetal growth in time and take corresponding intervention measures (19). Accurate assessment of gestational weeks can also provide more personalized pregnancy management advice for pregnant women. For example, for pregnant women with small gestational weeks but normal fetal growth indicators, monitoring can be stepped up to ensure that the fetus grows normally in subsequent gestational weeks; For pregnant women with large gestational weeks but slow fetal growth, further investigation of the causes and targeted treatment are needed (20).

The C-index of the nomogram model constructed in this study in the training set and the verification set was 0.858 and 0.835, respectively, indicating that the model had good discrimination (the high discrimination was defined as C-index > 0.8). The ROC curve showed a training set AUC of 0.858 and a validation set of 0.835, both higher than the predictive power of a single parameter (e.g., an AUC of approximately 0.75–0.80 for the uterine artery PI). This performance is also superior to previous studies focusing on ophthalmic artery parameters alone. For example, Abdel Azim et al. (21) reported an AUC of 0.78 (95% CI: 0.71–0.85) using ophthalmic artery PSV at 35–37 weeks for SGA prediction, which is lower than our model’s AUC of 0.858. The calibration curve showed good agreement between the predicted and actual values, and the *p* values tested by Hosmer–Lemeshow were all >0.05, indicating that the fitting degree of the model was ideal. Analysis of the decision curve further confirmed that the model had a high net clinical benefit when the threshold probability was between 0.10 and 0.80.

The nomogram integrates multiple risk factors through a visual scoring system, and clinicians can quickly calculate the risk probability of SGA based on the PSV ratio of pregnant women, PSV1, PSV2, PI1, PI2 and gestational week. For example, a pregnant woman with 37 weeks of gestation, PSV ratio 1.25, PSV1 = 35 cm/s, PSV2 = 28 cm/s, PI1 = 1.9, and PI2 = 1.7, had a total score of approximately 150 points, and a corresponding risk probability of approximately 60% for SGA. This quantitative assessment allows for the personalization of monitoring protocols (e.g., increased frequency of ultrasound examinations, fetal biophysical scores) or interventions (e.g., nutritional support, timely termination of pregnancy). Compared with previous models based on uterine and umbilical artery parameters, the model in this study included ophthalmic artery blood flow indicators, which reflected the fetal cerebrovascular state more comprehensively (22). A previous study involving 1,500 pregnant women showed a model AUC of 0.82 combining uterine artery PI with maternal BMI, whereas the model AUC in this study was higher (0.858). Compared with Anjos et al. (23), who used ophthalmic artery PI alone (AUC = 0.76) for fetal growth assessment, our model’s inclusion of PSV1, PSV2, and their ratio provides a more comprehensive hemodynamic profile. Additionally, Mansukhani et al. (24) developed a preeclampsia prediction model with ophthalmic artery Doppler (AUC = 0.80), highlighting that our model’s higher AUC for SGA suggests specific utility in fetal growth restriction rather than general pregnancy complications. In addition, in this study, PSV ratio was used as the core variable for the first time, thus expanding the application range of ophthalmic artery parameters.

We recommend integrating the nomogram into routine ultrasound workflows from ≥28 weeks of gestation, when clinical intervention is most effective. Standardized training for sonographers should emphasize PSV ratio measurement (intra-observer variability <5%), incorporating video tutorials on ophthalmic artery identification, quarterly proficiency tests (≥90% accuracy), and quality control protocols (e.g., insonation angle <20°) to minimize inter-observer variability (<8%). In high-risk populations (e.g., maternal hypertension), combining uterine artery Doppler with serum PlGF levels may further improve detection rates. Our preliminary data demonstrate the nomogram’s superiority over fetal abdominal circumference (AUC = 0.78), reducing false positives by 22% in obese women and false negatives by 18% in late-term pregnancies (≥37 weeks), with an ongoing prospective trial (*n* = 500) aiming for ≥15% improvement in SGA detection. For women with pre-pregnancy BMI ≥ 24 kg/m^2^, adjusting the PSV1 cutoff to >37 cm/s (1.3-fold higher in SGA cases) is advised, while those ≥35 years may benefit from combined PlGF screening (<32 pg./mL) to reduce false negatives by 15%. Sensitivity analysis supports a two-step protocol: initial PSV ratio screening (cutoff >1.2; sensitivity = 82%, specificity = 76%) followed by confirmatory PSV1/PI2 assessment, reducing unnecessary examinations by 30–40% and increasing PPV from 32 to 48%. Future studies should validate these findings across diverse cohorts and compare the nomogram head-to-head with current standards to quantify its clinical impact.

This study has the following limitations: First, the single-center retrospective design resulted in a small sample size (*n* = 421), the data sources were limited to specific time periods and regions, which might cause selection bias due to differences in medical habits and ethnic characteristics, and the generalization ability of the model was limited, making it difficult to be extended to different populations. Second, no multi-center external verification was performed, which was mainly limited by the difficulty in resource and time coordination, difficulty in data standardization (such as incomparable parameters due to differences in ultrasonic equipment model and operating specifications), and insufficient sample size (only 421 cases were sampled in total, and no more data could be split for verification). In addition, potential confounders such as placental growth factor (PGF), fetal cardiac function, and maternal inflammation indicators were not included in the study, which may weaken the prediction efficiency of the model. Furthermore, the study only included Han Chinese pregnant women, and the impact of ethnic diversity on model applicability remains unclear. Future multi-center studies should include participants of different ethnicities to enhance the generalizability of the model. Finally, there is a lack of long-term follow-up data for SGA neonates to assess the predictive value of the model for long-term complications such as neurodevelopmental abnormalities and metabolic disorders.

Future improvements should be made in the following areas: First, a multi-center large sample prospective study (with a recommended sample size of ≥500 cases) was conducted to verify the external validity of the model through the standardized ultrasound operating procedures, so as to ensure its applicability in different ethnic groups, gestational weeks and medical conditions. Second, a multi-modal prediction system was constructed by integrating multi-dimensional predictive indicators such as maternal serology (PGF, sFlt-1), placental ultrasound parameters (villous space flow) and fetal heart function (Tei index) to improve the prediction accuracy of SGA associated with placental insufficiency. Third, we traced the neonates with SGA to school age, analyzed the correlation between ophthalmic artery parameters and neurodevelopmental outcomes (such as intelligence and motor ability), and revealed the potential mechanisms of hemodynamic abnormalities and brain injury. Fourthly, digital decision tools based on nomograms (such as mobile phone applications or built-in software for ultrasound equipment) are developed, which combine artificial intelligence technology to optimize the model and integrate dynamic blood flow parameters to improve the timeliness of prediction.

In summary, the nomogram model constructed based on ophthalmic artery Doppler parameters in this study has high prediction efficiency for SGA, and provides a new tool for early clinical recognition of high-risk pregnant women. Among them, the combination of PSV ratio, PSV1, PSV2, PI1, PI2 and gestational week can significantly improve the accuracy of the prediction. However, the clinical transformation of the model still needs to be further verified, and the prediction system needs to be optimized in the future based on multidisciplinary data to provide evidence-based support for the accurate prevention and control of SGA.

## Data Availability

The raw data supporting the conclusions of this article will be made available by the authors, without undue reservation.
